# Functionalization of a Fully Integrated Electrophotonic Silicon Circuit for Biotin Sensing

**DOI:** 10.3390/bios13030399

**Published:** 2023-03-18

**Authors:** Oscar Pérez-Diaz, Denise Estrada-Wiese, Mariano Aceves-Mijares, Alfredo A. González-Fernández

**Affiliations:** National Institute for Astrophysics, Optics and Electronics (INAOE), Department of Electronics, Puebla 72000, Mexico

**Keywords:** electrophotonic (EPh) circuits, CMOS-compatible, functionalization, biotin sensing

## Abstract

Electrophotonic (EPh) circuits are novel systems where photons and electrons can be controlled simultaneously in the same integrated circuit, attaining the development of innovative sensors for different applications. In this work, we present a complementary metal-oxide-semiconductor (CMOS)-compatible EPh circuit for biotin sensing, in which a silicon-based light source is monolithically integrated. The device is composed of an integrated light source, a waveguide, and a p–n photodiode, which are all fabricated in the same chip. The functionalization of the waveguide’s surface was investigated to biotinylate the EPh system for potential biosensing applications. The modified surfaces were characterized by AFM, optical microscopy, and Raman spectroscopy, as well as by photoluminescence measurements. The changes on the waveguide’s surface due to functionalization and biotinylation translated into different photocurrent intensities detected in the photodiode, demonstrating the potential uses of the EPh circuit as a biosensor.

## 1. Introduction

The possibility to control and manipulate electrons and photons simultaneously in the same integrated silicon circuit (IC) introduces the novel concept of electrophotonics (EPh), where integrated photonics and electronics merge in a single IC. Given their combined nature, EPh systems present advantages by tackling the challenges of next-generation communication systems [1], biomedical [2], quantum, and sensing technologies [3]. Amongst its many uses, EPh presents promising applications in the sensing field, attaining the development of lab-on-a-chip systems where all the necessary components are monolithically integrated into the same platform. In addition, EPh has the added value of allowing us to exploit the already existing IC fabrication infrastructure for the miniaturization of the systems on a chip scale. Silicon-based photodetectors that rely on the absorption of visible to NIR light for optical-to-electrical conversion and integrated SiN waveguides are ideal candidates as basic components for EPh circuits [3,4]. These systems can be easily engineered to develop innovative sensors that use photonic mechanisms to detect different analytes [5,6]. Being able to determine if specific biomolecules are present in a sample is key for diverse healthcare applications, such as medical diagnosis [7], monitoring, and treatment. Over the last few years, many different silicon-based photonic biosensors that use diverse sensing techniques by means of the analyte’s interaction with light have been reported [2,8]. However, a light source is always needed for these devices to operate, which, in most cases, represents complicated and expensive integration of external or hybrid light emitters that always come with complications regarding alignment and insertion [9]. Moreover, non-integrated light detectors require special setup to function, making the devices impractical [9,10]. Thus, monolithically integrated light sources and photodetectors would be a great asset for practical and cost-effective optical biosensors.

In this work, we present for the first time, to our knowledge, the functionalization of an EPh circuit for biotin sensing where the light source, the waveguide (WG) with the bioreceptive region, and the photodetector are all contained in a single chip (7 × 4 mm^2^). The whole system is monolithically integrated into silicon and fabricated with CMOS-compatible materials and processes [11], with it being the first of its kind to be applied as a biosensor.

Biotinylation, also known as biotin labeling, serves as a protein-detection [12] or biomolecule-immobilization mechanism [13] since biotin covalently binds with very high affinity to specific proteins, such as streptavidin and avidin. Hence, biotin sensors are relevant for different tagging or detection applications. In the case of the device proposed here, the adhesion of biotin modifies the effective refractive index (n_eff_) in the material on top of the WG core, changing its characteristics of light propagation. The presence of other molecules further modifies n_eff_, and such changes result in different photocurrent values detected by the integrated photodiode. These changes can be contrasted to a reference measure, resulting in a potentially excellent sensor, as the close integration of all the electronic and photonic elements allows for the detection, identification, and correlation of small changes in light propagation as compared to non-integrated optical sensing approaches. The additional possibility of fluorescence from the analyzed species adds a further degree of specificity regarding the characteristics of the light after interacting with a specific substance.

To enable biotinylation on the EPh circuit, first, an analyte-specific functionalization of the WG surface is needed. This is achieved by coating the hydroxylated surface with 3-(aminopropyl) triethoxysilane (APTES), to which glutaraldehyde (GTA) is later covalently attached. Biotin is then deposited on the functionalized surface binding to the GTA molecules, finally reaching biotinylation. The surface is investigated on each step by microscopy, AFM, and Raman spectroscopy measurements, as well as photoluminescence (PL). Furthermore, the functionalized and biotinylated EPh system is evaluated through the changes in the detected photocurrent due to light–matter interactions with the molecules attached to the WG surface. Herein, we demonstrate the first fully integrated and functionalized EPh circuit for biotin sensing as a potential biomolecule sensor.

## 2. Materials and Methods

**Figure 1:**
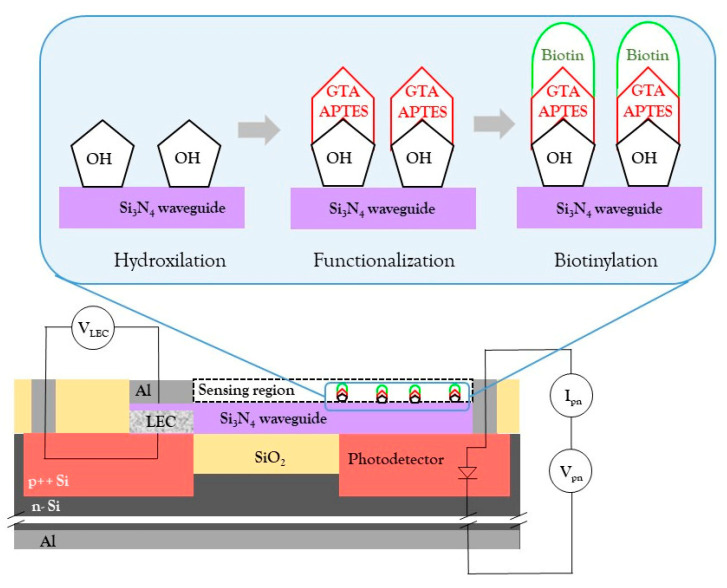
Cross-section of a schematic representation of the electrophotonic system (**bottom**) and the steps for activation, functionalization, and biotinylation of the sensing region on the surface of the Si_3_N_4_ waveguide core (**top**).

The EPh circuit consists of a Si-based light-emitting capacitor (LEC), a silicon nitride (Si_3_N_4_) waveguide core on a silicon dioxide (SiO_2_) cladding, and a p–n photodiode as the photodetector, all monolithically integrated into a Si substrate as schematized in Figure 1. This cutting-edge system was fabricated using all standard CMOS materials and procedures with no need for external light sources thanks to the LEC based on silicon-rich oxide (SRO) [4]. To operate the device, first, the LEC is biased by applying a voltage V_LEC_, producing light that is directly injected into the WG. The light emitted in the LEC is produced by electroluminescence in the SRO layer when inducing the current [14,15]. A portion of the propagating light in the WG (evanescent field) interacts with any substance placed on the surface of the WG, resulting in light-intensity changes. This modified transmitted light arriving to the photodetector depends on the nature of the deposited substance and its n_eff_ and is finally converted into detectable electrical current I_PN_ by the photodiode. Therefore, each different analyte has a different “transmission fingerprint” which could be related to specific values of the photocurrent. In addition, the possibility of fluorescent analytes (such as biotin) adds additional effects to the light interactions within the WG and the final intensity reaching the photodetector. So far, the viability of an EPh sensor based on a refractive scheme of the system, where fluorescence effects were not considered, has already been demonstrated through simulations in previous studies [6]. Further simulations considering other effects will be carried out in future work. A more detailed description of the EPh system and its fabrication process can be found in references [4,6].

For the study of the functionalized and biotinylated Si_3_N_4_ surfaces and their optical properties, we used Si wafers with a Si_3_N_4_ coating of the same characteristics as the WG core in the Eph system. Furthermore, the Si_3_N_4_ of the actual EPh devices was also functionalized and biotinylated following the same methodology.

The functionalization consisted of a sequence of steps starting with the activation of the hydroxyl (OH) groups on the Si_3_N_4_ surface, which are necessary for APTES bonding on the surface. The formation of the OH groups is commonly achieved by dipping the samples in a piranha solution [16], by applying heat treatment at 600 °C in an oxygen atmosphere [17], through oxygen plasma [18], or through an RCA cleaning process [19]. However, since the functionalization process was intended to be applied to the EPh circuit, it was necessary to follow a methodology that would not harm the device. For this reason, hydroxylation was achieved by submerging the samples into deionized water at 70 °C for 30 min, followed by heat treatment at 120 °C for 5 min in an N_2_ atmosphere [20]. The samples were then immersed in a 4% APTES (≥98%, Sigma-Adrich, Munich, Germany) solution in ethanol for a duration of 180 min to ensure full coverage of the surface. A rinsing and ultrasonic (1 min) process in ethyl alcohol (99.6%, J. T. Baker, Phillipsburg, NJ, USA) was performed to minimize non-specific attachment of APTES to the surface, followed by heat treatment at 110 °C in a N_2_ ambient temperature. Subsequently, the samples were immersed in a 4% GTA (25% in H_2_O, Sigma-Aldrich, Munich, Germany) solution in phosphate-buffered saline (PBS) (pH 7.4, Sigma-Adrich, Munich, Germany) for 120 min followed by a rinsing process in PBS and dried with N_2_. The APTES and GTA were deposited at room temperature in a N_2_ atmosphere to avoid further unwanted reactions [16,21,22]. Considering that this study is the first approach to the biotinylation of an EPh circuit, different concentrations of biotin were not explored. However, this subject will be addressed in the future, as it is relevant to evaluate, for instance, the influence of PL intensity on the detected I_photo_ and the detection limit of the system. Hence, following previous studies [23], biotinylation of the Si_3_N_4_ functionalized surfaces was achieved by submerging the samples in a 4 mM biotin (≥98%, Sigma-Adrich, Munich, Germany)–PBS solution for a duration of 120 min at room temperature. Finally, the samples were rinsed with PBS [23,24]. A schematic representation of the biotinylated WG surface on the EPh circuit is presented in the top part of Figure 1.

The Si_3_N_4_ surface was characterized after each of the previously explained steps, i.e., after hydroxylation (we call this surface non-functionalized), after functionalization (with APTES and GTA), and after biotinylation (attachment of biotin). The changes on the Si_3_N_4_ surface topography, due to the presence of the different molecules at each stage, were studied by optical microscopy (Leitz Dialux 20 microscope) and by atomic force microscopy (AFM) (Nanosurf easyScan in non-contact mode). Raman spectroscopy (WiTec alpha300R Confocal Raman Microscope) was used to confirm the bonding of the molecules. Furthermore, PL characterization (Horiba Jobin Yvon spectrometer model Fluoro-Max3) was performed to study the influence of the molecules on the Si_3_N_4_ surface. The PL measurements were carried out under controlled illumination conditions at ambient temperature, exciting the samples with UV light at 330 nm.

The evaluation of the capability of the EPh circuit to detect the presence of different molecules was performed by means of measurements and a comparison of photocurrent values produced by the photodiode. To measure the generated photocurrent I_pn (Vpn, VLEC)_, the photodetector was biased at a voltage V_pn_ = −20 V, while a voltage V_LEC_ was applied to the LEC (to produce light emission). However, it is necessary to eliminate any other current contribution (leakage current) and only consider the carriers generated by the injected light from the LEC. For this reason, instead of only using I_pn_, the current of the photodiode is biased with V_pn_ but, under dark conditions, I_pn (Vpn, 0)_ is also measured, i.e., when the LEC is turned off. Then, the reported photocurrent I_photo (Vpn, VLEC)_ can be obtained from the following equation:

I_photo (Vpn, VLEC)_ = I_pn (Vpn, VLEC)_ − I_pn (Vpn, 0)_ (1)

## 3. Results

### 3.1. Si_3_N_4_ Surface Characterization

**Figure 2:**
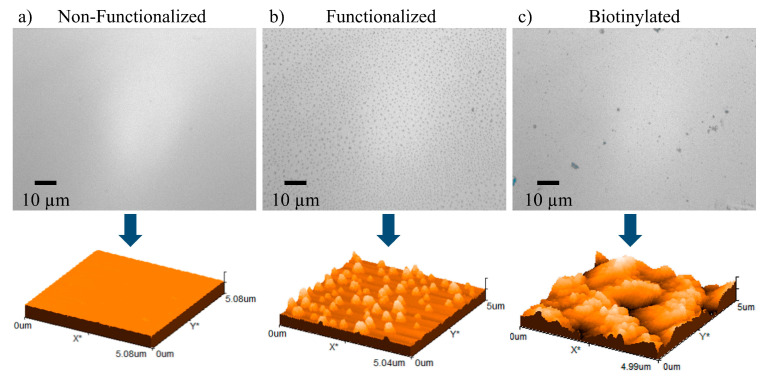
Optical microscopy (**top**) and AFM (**bottom**) images of the: (**a**) bare Si_3_N_4_ surface, (**b**) after APTES-GTA deposition, and (**c**) after biotin attachment.

The optical microscopy and AFM images of the Si_3_N_4_ surface are presented in Figure 2, with them first showing the flat bare Si_3_N_4_ surface (Figure 2a). After functionalization, through the deposition of APTES-GTA, it is possible to distinguish the presence of particles with spherical shapes (see Figure 2b), as reported in previous studies [13]. Figure 2c presents the surface of the samples after biotin attachment, where it is possible to observe bigger particles than before, which is associated with the presence of biotin on the functionalized surface. These results indicate a clear surface change as the different molecules were deposited.

### 3.2. Raman Spectroscopy

**Figure 3:**
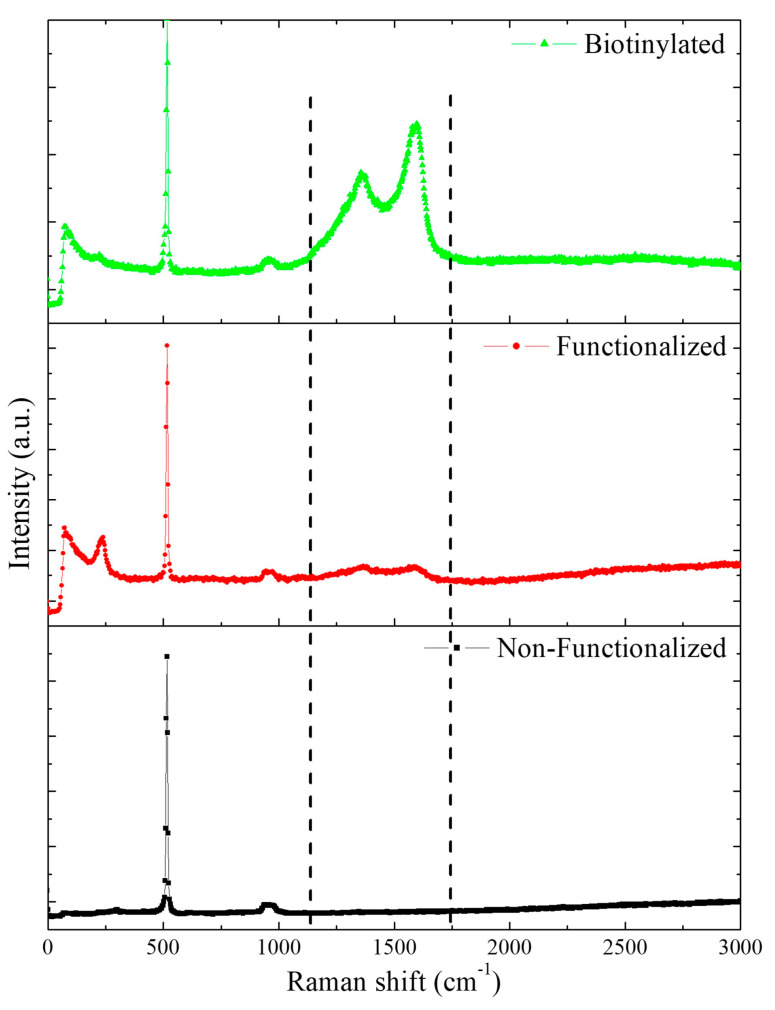
Raman spectra of the hydroxylated Si_3_N_4_ surface (black line), after functionalization (red line) and after biotinylation (green line).

The attachment of the different molecules after functionalization and biotinylation was further verified by Raman spectroscopy (using an excitation source at 532 nm) of the samples at each step, as presented in Figure 3. Here, the hydroxylated Si_3_N_4_ surface was taken as a reference spectrum. After APTES-GTA deposition it is possible to observe the appearance of signals in a range between 1100 cm^−1^ and 1800 cm^−1^, which could be related to the presence of NH_2_, CH_2,_ and CHO groups in the APTES and GTA molecules [25,26,27]. After biotin is deposited on the functionalized samples, an enhancement of the same peaks can be observed, since these previously mentioned groups are also present in the biotin molecules [28]. These results suggest that the biotin successfully attached to the functionalized surface.

### 3.3. Photoluminescence (PL)

**Figure 4:**
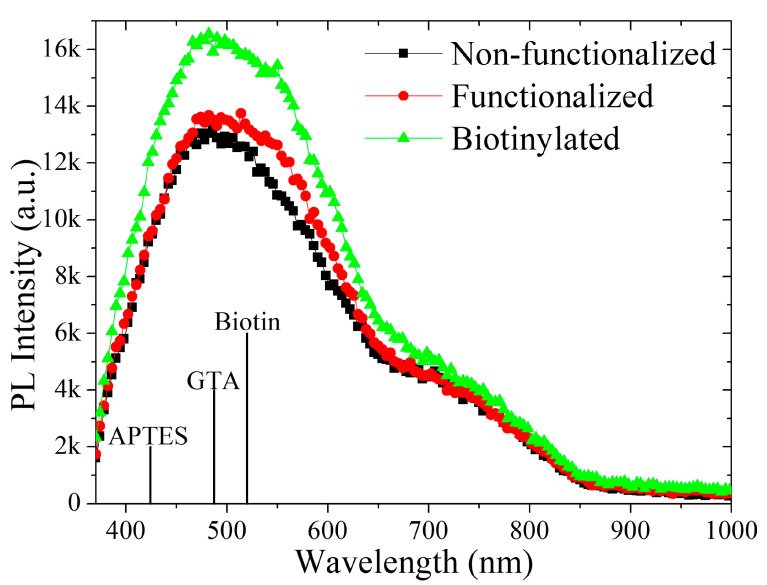
PL spectra for the Si_3_N_4_ surface (black line), after functionalization (red line) and after biotinylation (green line). The peak wavelengths of PL emission of the APTES, GTA, and biotin molecules are indicated on the wavelength axis [30,31,34].

Figure 4 shows the PL emission spectra of the samples when excited with a 330 nm wavelength light source. The contribution to photoemission of the non-functionalized Si_3_N_4_ surface presents a characteristic peak with a maximum intensity of around 500 nm, which agrees with the PL emission by Si_3_N_4_ recorded in [29]. The origin of this light emission can be attributed to a combination of transition states between the Si_3_N_4_ and oxide (produced during the hydroxylation process), nitride dangling bonds, and probably also surface defects induced by the OH groups [29,30]. A similar PL response is observed when the samples are functionalized, where only a slight rise in PL intensity is measured, as expected due to the presence of the APTES and GTA molecules [30,31]. The PL signal increases even further when biotin is deposited on the functionalized surface. This higher signal includes the contribution of luminescence by the biotin molecules themselves [32]. Here, as indicated in Figure 4, the characteristic wavelength at which biotin photoemits is 520 nm [31,33,34], which is closely around the maximum intensity peak (482 nm) of the PL signal of the biotinylated samples. These results, in addition to the previous findings, demonstrate the successful biotinylation of the Si_3_N_4_ surfaces.

### 3.4. Eph Circuit Measurements

**Figure 5:**
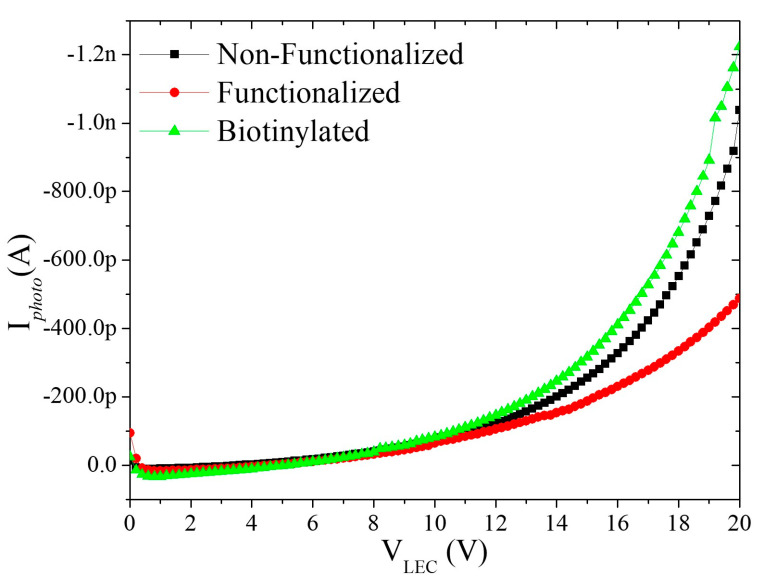
Measured photocurrent I_photo_ of the Eph circuit of the non-functionalized WG, after the functionalization process and after biotinylation.

The functionalization and biotinylation processes were applied on the WG of the Eph circuit following the same procedure as the prior characterized Si_3_N_4_ samples. Photocurrent measurements were performed at different stages of the process and the I_photo_ was calculated using equation 1 and setting V_pn_ = −20 V. In Figure 5, a representative measurement of the photocurrent I_photo_ tendency of an EPh device is presented. The shape of the curves is like those reported in [4]. It can be noted that, when the Eph circuit is non-functionalized, an I_photo_ of almost –1 nA is detected at V_LEC_ = 20 V, since the light passing through the WG has no interaction with any analyte on the surface other than air. However, when the WG is functionalized, I_photo_ decreases to –500 pA due to the interaction of the evanescent field with the APTES and GTA molecules. Moreover, as can be seen from the AFM images in Figure 2b, the functionalized surface shows particles of a few tens of nm in size distributed on the Si_3_N_4_ film, probably inducing light losses due to scattering effects. This results in lower light intensities arriving at the photodetector and hence lower I_photo_. On the other hand, once the biotin is attached to the functionalized surface, a significant I_photo_ increment is observed, which could be associated with a small contribution of biotin photoemission to the photocurrent [32]. More importantly, a possible increment of the refractive index of the biotinylated surface (APTES-GTA-biotin) compared to the non-functionalized surface (air), hence better confinement of the propagated light, could be the main cause for the enhanced photocurrent values [6].

## 4. Discussion

### 4.1. Functionalization of the EPh Circuit

In this study, the functionalization of the EPh circuit was validated to further analyze the attachment of biotin on the sensing region, i.e., the upper surface of the WG. The functionalization of the EPh system presented some challenges due to the nature of the circuit and its materials. For instance, a piranha solution or RCA cleaning is common for the hydroxylation procedure of the samples; here, however, to avoid etching and damaging of the Al contacts (Figure 1), a different approach needed to be followed. Hence, immersion in deionized water and annealing of the samples was shown to be a successful methodology to activate the OH groups required for the attachment of APTES and succeeding GTA. The following analysis through optical microscopy, AFM, and Raman spectra of the Si_3_N_4_ surfaces revealed the presence of the molecules in each of the steps leading to favorable biotinylation.

### 4.2. The EPh Sensor

The concept of the EPh circuit being used as a refractive index sensor evaluating the changes of the generated I_photo_ when different analytes are present on the WG surface has been proven in the past [6]. In this work the same principle has been further extended to the detection of molecules, such as biotin, that can be used for a diverse range of applications in the sensing area. The results show that, indeed, a change in the photocurrent was observed depending on the functionalization or biotinylation of the WG core, showing that the electrophotonic scheme can be applied to biosensing. The principle of operation of the biotin sensor presented here, in contrast to other detection schemes such as optical fiber or surface plasmon sensors [35], where an external light source is always required (usually a laser), is based on the detection of I_photo_ in a novel system where the light source (LEC) and the photodetector are fully integrated into the same circuit. However, the main drawback of the EPh system lies in the low light intensity emitted by the LEC translating into low current I_photo_ intensities in the pA–nA range. The experimental electroluminescence spectra of these SRO-based LECs have been measured to extend from blue to near-infrared, with two main peaks that vary with the V_LEC_ applied across the structure [29]. Much effort is being put into enhancing the light-emission intensities of the LECs by texturizing the substrate’s surface to promote larger electric field intensities around nanostructured sharp tips [36], but the extremely self-contained and close integration of the system also represents that it is possible to detect changes in photocurrent values much lower than in other systems, which also relaxes the requirement for high emission intensities [8]. As can be seen, there is still ongoing work to improve the monolithically integrated light emitters while maintaining the challenge of conserving CMOS compatibility.

### 4.3. Photocurrent

The intensity of the detected I_photo_ is directly correlated to the intensity of the light arriving at the photodiode. In a previous piece of work, it was shown experimentally and through simulations that the interaction of an analyte on the WG and the traveling light is strongly dependent on the refractive index of the respective analyte [6], as expected. In this work, the different attached molecules during functionalization and biotinylation also contribute to changes in the detected I_photo_, as can be seen in Figure 5. Refractive index measurements of the used molecules (GTA, APTES, and biotin) are not easy to obtain, since the commonly used spectroscopic ellipsometry method has complex difficulties due to the high roughness of the samples, as shown in Figure 2. Other techniques based on superficial plasmon resonance are also usually applied [37], but complicated devices are needed for the determination of the refractive index and this lies outside the scope of the present study. However, it has been demonstrated that when the refractive index contrast between the analyte and the lower SiO_2_ cladding (see Figure 1) is low, light is better confined in the WG, whereas if the refractive index contrast is high, dispersion within the WG increases causing higher losses [6]. Taking the latter into consideration for the highest intensity I_photo_ measurements obtained for the biotinylated device, it is reasonable to assume that the n_eff_ of the APTES-GTA-biotin layer is higher than the one of the APTES-GTA film since better confinement of the light resulted in higher I_photo_ values in the biotinylated system. Nonetheless, the functionalized samples also presented a highly uneven surface filled with particles (agglomerates of APTES-GTA) in sizes ranging a few tens of nm (Figure 2), which could lead to scattering effects and consequently large losses of the confined light thus presenting smaller I_photo_ values. In addition, another conceivable contribution to the higher I_photo_ measured on the biotinylated circuit might be due to the photoemission of the biotin molecules, as presented in Figure 4, adding to the intensity of the traveling light through the WG. Here, it is worth noticing that the power of the input light generated by the LEC is considerably lower (in the order of nW) than the light source used to excite the biotinylated samples during PL measurements (30 mW). Hence, we only assume that there might be a small contribution of the biotins PL emission to the light arriving to the photodetector, but a more thorough study needs to be carried out to fully understand this mechanism and the corresponding interactions within the WG.

## 5. Conclusions

In this work, it was proven that it is possible to functionalize a fully integrated EPh silicon circuit capable of sensing molecules such as biotin. This system has the capabilities to be scaled for biomolecule detection, such as for virus or antigen sensing for example. However, several challenges, such as molecule selectivity and the optimization of the Eph circuit itself, should be addressed first. Moreover, typical biosensor characteristics, including molecule affinity, sensitivity, and detection limits, are to be explored in the future. Here, a fully integrated Eph system, that can be used to detect different molecules, has been presented for the first time, proving its potential as a biosensor.

## Data Availability

Data are contained within the article.
